# The Impact of Obesity and a High-Fat Diet on Clinical and Immunological Features in Systemic Lupus Erythematosus

**DOI:** 10.3390/nu13020504

**Published:** 2021-02-04

**Authors:** Masanori Kono, Yasuo Nagafuchi, Hirofumi Shoda, Keishi Fujio

**Affiliations:** Department of Allergy and Rheumatology, Graduate School of Medicine, The University of Tokyo, Tokyo 113-8655, Japan; nagafuchi-tky@umin.ac.jp (Y.N.); shoda-tky@umin.ac.jp (H.S.); kfujio-tky@umin.ac.jp (K.F.)

**Keywords:** systemic lupus erythematosus, obesity, a high-fat diet, adipokines, leptin, adiponectin, vitamin D, microbiota, dysbiosis

## Abstract

Systemic lupus erythematosus (SLE) is a chronic autoimmune disease with multiple organ involvement predominantly affecting women of childbearing age. Environmental factors, as well as genetic predisposition, can cause immunological disturbances that manifest as SLE. A habitual high-fat diet and obesity have recently been reported to play a role in the pathogenesis of autoimmune diseases. The frequency of obesity is higher in patients with SLE than in general populations. Vitamin D and adipokines, such as leptin and adiponectin, are possible mediators connecting obesity and SLE. Serum leptin and adiponectin levels are elevated in patients with SLE and can impact innate and adaptive immunity. Vitamin D deficiency is commonly observed in SLE. Because vitamin D can modulate the functionality of various immune cells, we review vitamin D supplementation and its effects on the course of clinical disease in this work. We also discuss high-fat diets coinciding with alterations of the gut microbiome, or dysbiosis. Contingent upon dietary habits, microbiota can be conducive to the maintenance of immune homeostasis. A high-fat diet can give rise to dysbiosis, and patients who are affected by obesity and/or have SLE possess less diverse microbiota. Interestingly, a hypothesis about dysbiosis and the development of SLE has been suggested and reviewed here.

## 1. Introduction

Systemic lupus erythematosus (SLE) is an autoimmune disease that usually requires life-long treatment with glucocorticoids, hydroxychloroquine, and immunosuppressive agents. SLE is characterized by the appearance of a broad profile of autoantibodies such as anti-nuclear antibodies (ANA), anti-double strand DNA (anti-dsDNA) antibodies, anti-Ro antibodies, anti-La antibodies, and anti-Smith or Sm antibodies. Many of these antibodies develop before the clinical manifestation of SLE [1]. The deposition of immune complexes on affected organs and subsequent hypocomplementemia is also often observed. SLE mostly affects women of childbearing age [2], with a female to male ratio of approximately 9:1, which suggests female sex hormones may be implicated [3]. Clinical manifestations of SLE are diverse, and virtually no organ evades sequela. Typical presentations include hematological disorders—such as autoimmune hemolytic anemia, leukopenia, and thrombocytopenia—nephritis, arthritis, dermatitis, and neuropsychiatric involvement. Thus, SLE is considered a heterogeneous disease with various phenotypes, necessitating the adoption of tailored treatment [4].

Although not yet fully elucidated, a genetic predisposition and environmental factors, such as ultraviolet light exposure and smoking, contribute to the pathogenesis of SLE [5]. In essence, the pathogenesis of SLE can be summarized by the following four characteristics: (1) excessive apoptotic cell production and reduced disposal of apoptotic material with defective complement pathways; (2) nuclear antigen stimulation of innate immune cells such as plasmacytoid dendritic cells (pDCs) and subsequent aberrant production of type I interferon (IFN); (3) the breach of T cell tolerance accentuated by decreased regulatory T cells (Tregs) and, for example, their polarization to T_H_17 cells to alter T cell populations; and (4) a break in B cell tolerance, B cell activation, and autoantibody production [6]. Genome-wide association studies (GWAS) have reported more than 100 loci associated with SLE [7,8]. Some of the genes identified in GWAS are implicated in cellular pathways such as lymphocyte activation, IFN or Toll-like receptors, and immune complexes supporting waste clearance [6], all of which can contribute to the pathogenesis of SLE.

In the early 1950s, at the dawn of treatment for SLE, the 5-year survival rate was only 50% [9]; however, systemic glucocorticoids combined with hydroxychloroquine and immunosuppressive drugs such as mycophenolate mofetil, cyclophosphamide, and calcineurin inhibitors have dramatically improved the 10-year survival rate to more than 95%. Nevertheless, even today the risk of death in patients with SLE is approximately three times higher than in the general population of a comparable age group, with an exceptionally high risk of death posed by cardiovascular disease, infection, and kidney disease [10]. The long duration of high disease activity is a significant risk factor for cardiovascular events and contributes to mortality. However, glucocorticoids—drugs that are currently pivotal to the treatment of SLE—cause organ damage accrual in a dose-dependent manner [11,12,13,14,15]. Concerns also persist that immunosuppressive agents can cause serious infections, such as pneumocystis jirovecii pneumonia or progressive multifocal leukoencephalopathy caused by the John Cunningham (JC) virus. Thus, maintenance of the lowest possible level of disease activity under the lowest effective doses of glucocorticoid and immunosuppressive drugs is of vital importance. In this context, environmental factors other than pharmacological treatments also warrant close attention.

At present, awareness is growing that a high-fat diet and obesity may affect the course of autoimmune disease. The link between diet and the risk of developing autoimmune disorders was proposed half a century ago [16]. In the last two decades, much research has been undertaken with the aim of clarifying the relationships among diet, SLE development, and disease activity [17].

The impact of obesity on the pathogenesis of SLE and disease activity has attracted a lot of attention. The prevalence of obesity among patients with SLE is high, at approximately 30−40% [18,19,20]. A meta-analysis demonstrated that patients with SLE were more susceptible to metabolic syndrome compared with a healthy control population [21]. Metabolic syndrome is a cluster of concurrent metabolic factors closely linked to excessive weight and obesity that may include: abdominal obesity, high triglyceride levels, low high-density lipoprotein levels, hypertension, and impaired fasting glucose [16,22,23]. Long-term glucocorticoid therapy undoubtedly contributes to the development of obesity in patients with SLE. Recent research suggests that obesity itself might be a root cause of autoimmune diseases, high disease activity, and a poor prognosis [24], although this remains controversial. Because a high-fat diet can cause obesity, the relationship between a high-fat diet and SLE should also be considered.

In this review, we focus on how a high-fat diet and obesity can exacerbate SLE. We begin by briefly summarizing the pathogenesis of SLE to explain how immunological disturbances can be attributed to some extent to a high-fat diet and obesity and the relationship of causality to SLE.

## 2. The Pathogenesis of SLE

The pathogenesis of SLE is quite complex and not yet fully understood but can be put into better context by further addressing the four mechanisms that appear to underpin its development.

First, an imbalance between apoptotic cell production and the disposal of apoptotic material [6] can be caused by an increase in apoptotic cells as a result of environmental factors such as ultraviolet light and infections. Cellular death can occur via neutrophils that release nuclear antigens called neutrophil extracellular traps (NETs), and the neutrophils of patients with SLE tend to exhibit an increased propensity for NETs or NETosis as the process is known [25]. Defects in the removal of NETs and the abnormal induction of NETosis have been reported in SLE [26]. A defect in the complement pathway, essential for opsonization and clearance of immune complexes and apoptotic cells, is involved in the development of SLE [4]. Deficiencies in classical complement pathway genes are strongly associated with an increased susceptibility for SLE [27]. In addition, gene mutations involved in DNA processing during apoptosis cause lupus-like systemic autoimmunity [4]. Thus, in patients with SLE, increased nuclear antigens and decreased clearance cause a net increase in nuclear autoantigens.

Second, nuclear antigens stimulate intracellular sensors such as Toll-like receptors (TLRs) and cytosolic nucleic acid sensors, such as the stimulator of IFN genes (STING). Specifically, TLR7, which senses single-stranded RNA and TLR9, recognizes unmethylated CpG motifs [28]. The stimulation of these sensors leads to IFN-α production from immune cells, including pDCs.

Third, the loss of T cell tolerance and an increase of a pathogenic helper T cell subset is associated with the pathogenesis of SLE. An increase in T_H_17 cells and a decrease in Treg cells is reported in humans with SLE [29]. The immune system is equipped with an immune tolerance mechanism that distinguishes between self and non-self and is tuned so as not to elicit an autoimmune response. For example, during negative selection, self-reactive T cells are eliminated during differentiation in the thymus. Recent studies have shown that this negative selection is imperfect, and some of the autoreactive T cells that escape negative selection are regulated by Tregs [30]. Decreased Treg function, decreased levels of interleukin (IL)-2, essential for Treg cell development and function [6,31], and pathogenic T_H_17 cells are reportedly implicated in SLE. Increased levels of serum IL-17 [31] and T_H_17 cells can infiltrate the kidneys of patients with lupus nephritis [6]. In humans with SLE, low-dose IL2 treatment may be useful for the restoration of Tregs and to reduce T_H_17 cells and follicular T helper (Tfh) cells [32].

Lastly, a break in B cell tolerance, B cell activation, and autoantibody production play a role in the pathogenesis of SLE. B cell activation and autoantibody production is accelerated by B-cell activating factor (BAFF), which is upregulated in SLE [6]. Belimumab, a monoclonal antibody targeting BAFF, has been used to treat SLE [33] via the reduction of anti-dsDNA antibody titers, elevation of complement levels, and alleviation of musculoskeletal and dermatological manifestations. Also, TLR signal transduction, the activation of B cell support by Tfh cells, contributes to the breach of B cell tolerance [6].

In summary, increased nuclear antigens, the stimulation of intracellular sensors such as TLRs and subsequent production of IFN-α from immune cells, the polarization of helper T cells to T_H_17 cells, and the breach of T cell and B cell tolerance are all implicated in the pathogenesis of SLE.

## 3. The Conceivable Link between Obesity and SLE

Obesity is defined as a body mass index (BMI) over 30 kg/m^2^ and corresponds to excessive body fat in the form of adipose tissue. It is now well known that obesity is characterized by a state of chronic low-grade inflammation; that is, obesity induces pro-inflammatory cytokines such as tumor necrosis factor-alpha (TNF-α) and IL-6 [17]. The connection between obesity and autoimmunity has been vigorously investigated. In addition to TNF-α and IL-6, adipokines such as leptin and adiponectin are produced by white adipose tissue (WAT) [24]. Adipose tissue is classified as white or brown (BAT); the former is an energy reservoir and BAT is responsible for thermogenesis; although BAT is not retained in adulthood [34]. Adipokines could be central to connecting obesity and autoimmunity and should be reviewed in detail.

Another possible explanation for the connection between obesity and SLE is a vitamin D deficiency. Vitamin D plays a role in the maintenance of innate and adaptive immunity. The prevalence of a vitamin D deficiency is higher in patients with obesity than in eutrophic controls [35]. Patients with SLE also tend to be vitamin D deficient [36]. Although it remains controversial, vitamin D is often considered to be a potential factor that connects obesity with SLE.

The direct relationship between a high-fat diet and SLE has been investigated. Dysbiosis, or alteration of the gut microbiome, can be caused by a high-fat diet regardless of obesity in a murine model [37]. Microbiota in the human gut contributes to the maintenance of immune homeostasis [38]. Dysfunction between the microbiome and the host is associated with various diseases such as autoimmune diseases, infections, and cancer [39]. A high-fat diet reportedly reduces the diversity of fecal microbiota, which likely influences the host immune system [40]. Similarly, fecal microbiota compositions from primary Sjögren’s syndrome and SLE showed a decreased bacterial diversity [41]. Thus, a high-fat diet can cause dysbiosis, which could contribute to the pathogenesis of SLE, particularly as it relates to adipokines and vitamin D.

### 3.1. Adipokines

#### 3.1.1. Leptin

Leptin was the first adipokine cloned in 1994 [42]. Leptin is a 16 kDa nonglycosylated polypeptide hormone encoded by the obese (ob) gene, the murine homolog of the human LEP gene [24,43]. Leptin is mainly produced by WAT and exerts its function by acting on leptin receptors. Leptin suppresses appetite by interacting with leptin receptors in the hypothalamic nuclei and enhances energy expenditure by regulating glucose and lipid metabolisms [24]. Circulating leptin positively correlates with adipocyte size and the body adipose mass [24,44]. Because leptin receptors are widely distributed beyond the hypothalamus in various organs including the kidneys, lungs, and adrenal glands [45], leptin is considered to possess pleiotropic functions. Of note, leptin can modulate the immune system because its receptors are also expressed on immune cells. Recently, more evidence has emerged to suggest that leptin is implicated in the pathogenesis of SLE [43].

Many researchers indicate that serum/plasma leptin is elevated in patients with SLE [46,47,48,49]. In contrast, most of the reports suggest that leptin levels are not correlated with disease activity [46,47,48,49]; although the relationship between the SLE Disease Activity Index (SLEDAI) and leptin remains controversial. High concentrations of leptin have been shown to significantly increase the risk of subclinical atherosclerosis in patients with SLE [47]. Taken together, it can be hypothesized that leptin in serum/plasma, even when independent of SLE disease activity, may contribute to damage accrual in SLE.

There are several hypotheses as to why concentrations of leptin in serum are linked to SLE. In leptin-deficient (ob/ob) mice, leptin deficiency protected against the development of autoantibodies and renal disease, with an increase in Tregs after treatment with the SLE-inducing agent pristane [50]. In an in vitro study, leptin enhanced effector T cell responses, promoted the presentation of self-antigens to T cells, and inhibited Treg activity [50]. Consistently, fasting was found to induce hypoleptinemia and expansion of Tregs in lupus-prone mice [51]. In a mouse model of leptin-deficient lupus, Tregs were increased until supplemental leptin decreased them [52]. In line with those reports, leptin in serum and Tregs as a percentage of total CD4^+^ T cells were negatively correlated in humans with SLE [53]. Leptin has also been reported to induce retinoic acid receptor-related orphan receptor gamma-t (RORγt) expression and expand T_H_17 cells in lupus-prone mice [54]. Leptin-deficient lupus mice exhibited decreased T_H_17 cells, anti-DNA antibody titers, and ameliorated nephritis [52]. Thus, leptin has the potential to affect the course of SLE by modulating T_H_17/Treg balance.

#### 3.1.2. Adiponectin

The first report on adiponectin was published in 1995 [55]. Adiponectin is mainly synthesized by WAT [43] and structurally similar to the subunits of complement factor C1q [55]. Contrary to leptin, adiponectin is considered anti-inflammatory; adiponectin vigorously protects against pathological events in various cells by inhibiting cell death, suppressing inflammation, and promoting cell survival [56]. Low levels of adiponectin are linked to type 2 diabetes mellitus and coronary artery disease [43]. Adiponectin has a protective effect on the vascular walls; it inhibits the expression of adhesion molecules induced by TNF-α, resulting in a decrease of monocyte adhesion to endothelial cells [24]. Adiponectin also inhibits macrophage transformation to foam cells [24], which positively impacts artherosclerosis.

In contrast to leptin, circulating concentrations of adiponectin are low in patients who are affected by obesity, partly because of the presence of a feedback inhibition process [57]. TNF-α for example, which is increased in patients with obesity, has the potential to inhibit adiponectin production in human adipocytes in vitro while leptin production remained intact [58].

Interestingly, many reports suggest that adiponectin in serum increases in patients with SLE [43], contrary to the elevation of leptin in SLE. A recent meta-analysis that analyzed the data of 782 patients from eight studies showed that the patients with SLE exhibited higher concentrations of adiponectin in serum than control subjects [59]. Subgroup analyses in the same report revealed that patients with SLE and a BMI of 25 kg/m^2^ or more had higher concentrations of adiponectin in serum compared with controls [59]. Considering that adiponectin tends to be relatively low in patients who are affected by obesity, these results may seem contradictory and require further investigation.

Levels of adiponectin do not correlate with disease activity [24,59]. However, there is a report that adiponectin in serum is higher in those patients with SLE who have lupus nephritis as opposed to those without nephritis [60]. Moreover, the severity of proteinuria was correlated with adiponectin in serum [60].

Adiponectin also has an anti-inflammatory effect on immune cells. Adiponectin increases Tregs and inhibits T-cell and B-cell activation and proliferation [24]. Adiponectin also regulates innate immunity; it induces anti-inflammatory cytokines such as IL-1 and IL-10, while at the same time, it reduces the secretion of TNF-α and IL-6 from antigen-presenting cells, including macrophages and dendritic cells (DCs) [24,43]. However, these anti-inflammatory properties of adiponectin do not explain why it is typically high in patients with SLE. Further studies are required to better understand the function of adiponectin in patients with SLE.

### 3.2. Vitamin D

Obesity has been shown to be linked to vitamin D deficiency, which is defined as <20 ng/mL of 25hydroxyvitamin D [61]. In a meta-analysis, the prevalence of a vitamin D deficiency in subjects who were affected by obesity was 35% higher than in eutrophic subjects [35].

The high global prevalence of vitamin D deficiencies, affecting up to two-thirds of the population with SLE [62], has been noted in many reports and meta-analyses [36,63,64,65]. It has been suggested that SLE is implicated in vitamin D deficiencies and, although sun exposure is a primary source of vitamin D, patients are usually advised to avoid it; a lack of this hormone remains a potential risk factor for the exacerbation of SLE. A higher prevalence of chronic kidney disease due to lupus nephritis may also be a cause. The risk factors for severe vitamin D deficiency (25-hydroxyvitamin D below 10 ng/mL) are the presence of photosensitivity and renal disease [62]. The administration of glucocorticoids may be another factor that increases susceptibility to a vitamin D deficiency in SLE [66]. Long-term glucocorticoid administration causes a reduction of intestinal vitamin D absorption and enhances the catabolism of vitamin D via an increase in CYP24A1 activity [67].

Although vitamin D receptor (VDR) polymorphisms have been associated with a high risk of SLE in a meta-analysis [68], the GWAS have not shown a corresponding association [69]. The active form of vitamin D, 1.25(OH)_2_D (calcitriol), binds to VDR, exerting various biological effects. Low levels of vitamin D are reported to precede the diagnosis of SLE and predict disease progression [66]. In healthy individuals positive for ANAs, the concentration of vitamin D is lower than for those who are negative [70], suggesting that vitamin D may play a role in the pathogenesis of SLE.

Vitamin D has attracted increasing attention from clinicians, given that it not only plays an essential role in bone mineral homeostasis but also modulates innate and adaptive immunity. VDRs are expressed in multiple immune cells such as macrophages, DCs, T cells, B cells [71], and neutrophils [72]. The treatment of murine DCs with calcitriol in vitro reduced the production of IL12 without inducing the production of TNF-α [73]. Calcitriol-treated DCs demonstrated resistance to maturation [73], indicating that calcitriol had the potential to inhibit T cell activation by mature DCs in an antigen-specific manner. Similarly, calcitriol acts on human macrophages stimulated with lipopolysaccharide to reduce the production of the pro-inflammatory cytokines IL-6 and TNF-α [74]. Vitamin D also impacts adaptive immune cells, such as B cells and CD4^+^ T cells. Many reports suggest that calcitriol inhibits B cell proliferation, immunoglobulin class switching, and antibody production [75]. Calcitriol also inhibits T_H_1 cytokine production, T_H_17 cell differentiation and activation, and IL-17 production, and induces Treg cell differentiation [75]. The decrease in Tregs and the increase in T_H_17 cells, together with B cell activation, are implicated in the pathogenesis of SLE and it seems likely that a vitamin D deficiency is involved in the development of SLE.

The results to date are contradictory with regard to whether a vitamin D deficiency is linked to high disease activity in patients with SLE [75,76]. Owing to the heterogeneous nature of SLE, many factors may affect the course of the disease.

If a vitamin D deficiency contributes to the cause of SLE, it seems logical to consider what impact vitamin D supplementation may have in terms of disease activity. The results of relevant studies are summarized in Table 1. The most extensive study addressing this topic emanated from a prospective cohort study by Petri et al. that included 1006 patients with SLE, of whom 76% had 25-hydroxyvitamin D levels below 40 ng/mL (low levels of vitamin D) [77]. The study showed that an increase in the levels of 25hydroxyvitamin D was associated with a modest decrease in disease activity in those with initially low levels of vitamin D [77]. The beneficial effect was not observed in those with a 25-hydroxyvitamin D concentration higher than 40 ng/mL [77]. No significant association between vitamin D levels and anti-dsDNA titers or C-reactive protein was observed [77]. One RCT of patients with juvenile-onset SLE demonstrated a statistically significant improvement in the SLEDAI score in the vitamin D supplemented group versus the placebo group [78], although the change in the score was small (SLEDAI score 0 vs. +1, respectively). On the contrary, two other RCTs revealed no beneficial effect of vitamin D supplementation on SLE disease activity [79,80]. Also, Vitamin D supplementation did not modify the interferon signature response [79]. One possible explanation for these contradictory results is that schedules and dosages of vitamin D supplementation were highly variable [67].

In summary, although current studies have not concluded that vitamin D supplementation contributes to a reduction in SLE disease activity, some studies reveal a beneficial effect. If patient selection strategies, doses, and the duration of vitamin D supplementation are to be optimized, patients with SLE will certainly benefit. Accordingly, although official management guidelines for SLE, such as the European League Against Rheumatism recommendations [81], do not mention vitamin D supplementation, vitamin D supplementation is recommended for patients with SLE [67].

### 3.3. High-Fat Diets and Dysbiosis

One of the plausible hypotheses linking a high-fat diet to immunological modifications relates to dysbiosis. An enormous number of microorganisms reside in the gastrointestinal (GI) tract [82]. Within the GI tract, the immune system is confronted with various antigens presented by intestinal microorganisms and food such that the innate and adaptive immune systems must play a role in maintaining the balance between tolerance to commensal microorganisms and reactions mounted against pathogens [82,83]. Innate immunity is contingent upon the expression of pattern recognition receptors (PRRs) on intestinal epithelial cells where signal transduction in relation to microbial recognition is essential for maintaining the intestinal epithelial barrier [82]. Recently discovered innate lymphoid cells (ILCs) of the intestine, particularly the ILC3 type, are reportedly regulated by microbiota [82]. Briefly, ILC3 is abundant in the mucosal lamina propria and produces IL-22, which acts on intestinal epithelial cells to stimulate the production of antimicrobial peptides, thereby providing protection against bacterial, fungal, viral, and parasitic infections [84,85]. Also, ILC3 can process and present microbial antigens to CD4^+^ T cells, limiting commensal bacteria-specific CD4^+^ T-cell responses [86]. Adaptive immunity is important because it focuses on CD4^+^ T cells, particularly Tregs and T_H_17 cells in the intestinal lamina propria. T_H_17 cells are more abundant than other T_H_ cell subsets in the GI tract [83]. T_H_17 cells produce IL-17 and IL-22, which contribute to defenses against fungal and bacterial infections [83]. An excessive T_H_17 response can be suppressed locally by the luminal disposal of T_H_17 cells or differentiation of pathogenic T_H_17 cells to non-pathogenic T_H_17 cells [16]. T_H_17 cells are reported to play a pathogenic role in SLE [6] and various other autoimmune diseases. Microbial antigens captured and presented by dendritic cells lead to the differentiation of commensal specific Tregs [87]. T_H_17/Treg balance is considered to be modulated by the gut microbiome [16].

Many effects of a high-fat diet have been investigated thus far. A high-fat diet has been shown to cause dysbiosis as a result of a lower ratio of Bacteroidetes to Firmicutes [83]. The dysbiosis caused by consuming a high-fat diet occurred even in the absence of obesity in a murine model [37]. The diversity of fecal microbiota, which may influence the host’s immune system, was reduced with exposure to a high-fat diet [40]. Furthermore, intestinal permeability drastically increased, and the expression of genes for tight junction proteins was reduced in mice that were fed a high-fat diet [88]. TLR7 on DCs were induced by a high-fat diet, which led to the exacerbation of SLE in TLR8-deficient mice [89]. A change in microbiota that alters the intestinal structure and increases intestinal permeability may enhance the translocation of microbes and antigens [40], which, together with the aberrant expression of PRRs such as TLR7, could stimulate the innate and adaptive immune systems.

Fecal microbiota composition from patients with primary Sjögren’s syndrome and SLE showed decreased bacterial diversity [41]. In patients with SLE, a higher ratio of Bacteroidetes to Firmicutes was observed [90], which was contrary to what occurred in patients affected by obesity [91]. However, controversy persists about whether dysbiosis causes or contributes to the pathogenesis of SLE [92]. Given that enteritis is sometimes observed, it is not a frequently involved organ in patients with SLE, and so the causal relationship between dysbiosis and SLE seems unexpected [38].

In 2018, Vieira et al. reported that the translocation of *Enterococcus gallinarum*—from the intestinal tract to the liver and systemic tissues—causes lupus-like disease in a lupus-prone murine model [93]. *E. gallinarum* RNA is a potential TLR7/8 ligand and induces type Ⅰ IFN from hepatocytes and DCs [93]. As noted previously, TLR7 and IFN are implicated in the pathogenesis of SLE. *E. gallinarum* has down-regulated molecules that function at the intestinal barrier [93]. Aryl hydrocarbon receptor signaling is enhanced by *E. gallinarum*, leading to the induction of T_H_17 cells and Tfh cells [93]. Interestingly, vaccination against *E. gallinarum* reduced autoantibody titers and improved survival in lupus-prone mice [93]. Strikingly, liver biopsies from patients with SLE were positive for *E. gallinarum*, implying that a specific gut pathobiont such as *E. gallinarum* may be implicated in the development of SLE.

Greiling et al. reported in 2018 that commensal Ro60 orthologs could trigger autoimmunity in SLE [94]. Anti-Ro antibodies are found in approximately 50% of patients with SLE [1] and are considered pathogenic. The Ro60 protein, a ring-shaped RNA binding protein forming ribonucleoprotein complexes, is highly evolutionarily conserved [94]. The authors found that Ro60-containing bacteria could activate human Ro60-specific memory CD4^+^ T cells, and furthermore, colonization with Ro60 ortholog-containing gut microbiota led to the development of lupus-like disease in germ-free mice [94]. These results highlight the importance of gut microbiota in the development of SLE.

## 4. Conclusions

**Figure 1:**
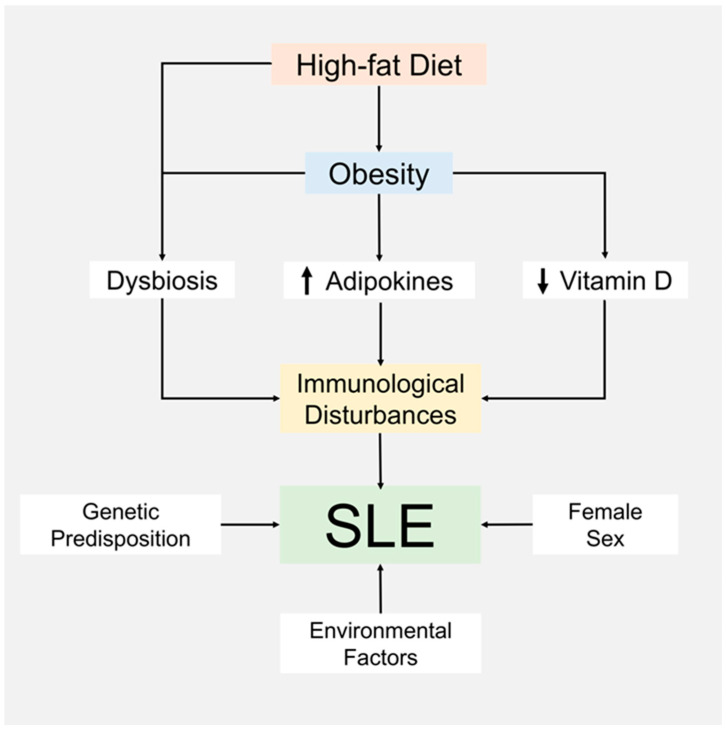
Suggested hypothesis about the connections between a high-fat diet, obesity, and the pathogenesis of SLE.

Although it is still controversial, mounting evidence suggests that obesity and a high-fat diet are linked to SLE, given that the frequency of obesity in patients with SLE is much higher than in healthy populations. This review summarizes possible mechanisms that connect obesity with SLE, including a vitamin D deficiency and an increase in adipokines, such as leptin and adiponectin, all of which are frequently observed in individuals with obesity and patients with SLE. We introduce the concept that a high-fat diet can cause dysbiosis of gut microbiota, which may be involved in the pathogenesis of SLE. We also note that a genetic predisposition and female sex continue to be explanatory factors for the development of SLE. The suggested hypothesis intersecting mechanisms for obesity, a high-fat diet, and SLE are summarized in Figure 1. SLE is a heterogeneous disease with variable disease activity and organ involvement that can require different approaches to treatments. The nature of SLE makes it challenging to accumulate definitive evidence showing how obesity, adipokines, vitamin D, a high-fat diet, and dysbiosis contribute to outcomes in clinically meaningful terms. The suggested hypotheses need to be verified by further clinical research.

## Figures and Tables

**Table 1:** Summary of trials on the effects of vitamin D supplementation on disease activity in SLE.

Study (Year)	Number of SLE Patients	Study Design	Reduction in Disease Activity	Results
Petri et al. (2013) [77]	1006	Prospective cohort	Statistically significant *	A 20 unit increase in 25(OH)D was associated with a 0.22 decrease in SELENA-SLEDAI ^1^
Aranow et al. (2015) [79]	57	RCT	NS	No effect on the expression of IFN signature genes
Lima et al. (2016) [78]	40	RCT	Statistically significant *	SLEDAI score change in the vitamin D supplemented group vs. the placebo group (0 vs. +1)
Karimzadeh et al. (2017) [80]	90	RCT	NS	Mean SLEDAI score before versus after the administration of vitamin D (3.09 vs. 1.62 ± 1.25, respectively)

25(OH)D: 25-hydroxyvitamin D; IFN: interferon; NS: not significant; RCT: randomized controlled trial; SELENA: Safety of Estrogens in Systemic Lupus Erythematosus National Assessment; SLEDAI; Systemic Lupus Erythematosus Disease Activity Index. ^1^ SELENA-SLEDAI: modified version of SLEDAI. * Note that the authors of those studies admit that further research is required to assess the clinical significance of the results.

## Data Availability

Data sharing not applicable.
